# Randomized trial to compare the efficacy and toxicity of cyclophosphamide, methotrexate and 5-fluorouracil (CMF) with methotrexate mitoxantrone (MM) in advanced carcinoma of the breast

**DOI:** 10.1038/sj.bjc.6990694

**Published:** 1999-09

**Authors:** C Harper-Wynne, J English, L Meyer, M Bower, C Archer, H D Sinnett, C Lowdell, R C Coombes

**Affiliations:** Department of Medical Oncology, Cancer Research Campaign Laboratories, Imperial College School of Medicine, Charing Cross Hospital, Fulham Palace Road, London, W6 8RF, UK; Clinical Trials and Statistics Unit, Block D, Section of Epidemiology, The Institute for Cancer Research, Sutton, Surrey, SM2 5NG, UK; Department of Surgery Department of Radiotherapy, Charing Cross Hospital, Fulham Palace Road, London, W6 8RF, UK

**Keywords:** breast cancer, chemotherapy, metastases, mitoxantrone, methotrexate

## Abstract

One hundred and sixteen patients with locally advanced or metastatic breast cancer were randomized to receive CMF (cyclophosphamide 600 mg m^−2^ day 1 and 8 i.v., 5-fluorouracil 600 mg m^−2^ day 1 and 8 i.v.,, methotrexate 40 mg m^−2^ day 1 and 8 i.v., monthly for 6 cycles) or MM (methotrexate 30 mg m^−2^, mitoxantrone 6.5 mg m^−2^, both i.v. day 1 3-weekly for 8 cycles) as first line treatment with chemotherapy. Objective responses occurred in 17 patients out of 58 (29%) who received CMF and nine out of 58 (15%) who received MM; 95% confidence interval for difference in response rates (–1%–29%), *P* = 0.07. No statistically significant differences were seen in overall survival or time to progression between the two regimes although a tendency towards a shorter progression time on the MM regime must be acknowledged. There was, however, significantly reduced haematological toxicity (*P* < 0.001) and alopecia (*P* < 0.001) and fewer dose reductions and delays in patients randomized to MM. No statistically significant differences were seen between the two regimes in terms of quality of life (QOL). However, some association between QOL and toxicity was apparent overall with pooled QOL estimates tending to indicate a worsening in psychological state with increasing maximum toxicity over treatment. Despite the fact that results surrounding response rates and time to progression did not reach statistical significance, their possible compatibility with an improved outcome on CMF treatment must be borne in mind. However, MM is a well-tolerated regimen with fewer side-effects than CMF, which with careful patient management and follow-up, therefore, may merit consideration as a first-line treatment to palliate patients with metastatic breast cancer who are infirm or elderly. © 1999 Cancer Research Campaign


British Journal of Cancer (1999) **81**(2), 316–322
© 1999 Cancer Research Campaign
Article no. bjoc.1999.0694

### Randomized trial to compare the efficacy and toxicity of cyclophosphamide, methotrexate and 5-fluorouracil (CMF) with methotrexate mitoxantrone (MM) in advanced carcinoma of the breast

**C Harper-Wynne[1], J English[1], L Meyer[2], M Bower[1], C Archer[1], HD Sinnett[3], C Lowdell[4] and RC Coombes[1]**

[1]Department of Medical Oncology, Cancer Research Campaign Laboratories, Imperial College School of Medicine, Charing Cross Hospital, Fulham Palace Road, London W6 8RF, UK; [2]Clinical Trials and Statistics Unit, Block D, Section of Epidemiology, The Institute for Cancer Research, Sutton, Surrey SM2 5NG, UK; [3]Department of Surgery and [4]Department of Radiotherapy, Charing Cross Hospital, Fulham Palace Road, London W6 8RF, UK

**Summary** One hundred and sixteen patients with locally advanced or metastatic breast cancer were randomized to receive CMF (cyclophosphamide 600 mg $m^{-2}$ day 1 and 8 i.v., 5-fluorouracil 600 mg $m^{-2}$ day 1 and 8 i.v.,, methotrexate 40 mg $m^{-2}$ day 1 and 8 i.v., monthly for 6 cycles) or MM (methotrexate 30 mg $m^{-2}$, mitoxantrone 6.5 mg $m^{-2}$, both i.v. day 1 3-weekly for 8 cycles) as first line treatment with chemotherapy. Objective responses occurred in 17 patients out of 58 (29%) who received CMF and nine out of 58 (15%) who received MM; 95% confidence interval for difference in response rates (–1%–29%), $P = 0.07$. No statistically significant differences were seen in overall survival or time to progression between the two regimes although a tendency towards a shorter progression time on the MM regime must be acknowledged. There was, however, significantly reduced haematological toxicity ($P < 0.001$) and alopecia ($P < 0.001$) and fewer dose reductions and delays in patients randomized to MM. No statistically significant differences were seen between the two regimes in terms of quality of life (QOL). However, some association between QOL and toxicity was apparent overall with pooled QOL estimates tending to indicate a worsening in psychological state with increasing maximum toxicity over treatment. Despite the fact that results surrounding response rates and time to progression did not reach statistical significance, their possible compatibility with an improved outcome on CMF treatment must be borne in mind. However, MM is a well-tolerated regimen with fewer side-effects than CMF, which with careful patient management and follow-up, therefore, may merit consideration as a first-line treatment to palliate patients with metastatic breast cancer who are infirm or elderly.

© 1999 Cancer Research Campaign

**Keywords:** breast cancer; chemotherapy; metastases; mitoxantrone; methotrexate

Since Greenspan and Canellos evaluated combination chemotherapy for metastatic breast cancer there have been continued changes to the various combinations used to treat advanced breast cancer (Greenspan, 1966; Canellos et al, 1976). Combination chemotherapy for metastatic breast cancer can achieve tumour response rates of between 30 and 60% (Mouridsen, 1992; Honig, 1996). However, improvements in long-term remissions and overall survival have been difficult to achieve and thus the challenge in advanced breast cancer is to develop regimens that have low subjective toxicity whilst maintaining clinical efficacy.

Cyclophosphamide, methotrexate and 5-fluorouracil (CMF) in its range of schedules is often considered a standard regime for first-line treatment of metastatic breast cancer with response rates between 30 and 60% (Bull et al, 1978; Tormey et al, 1982; Aisner et al, 1987; Cummings et al, 1995). Methotrexate, mitoxantrone and mitomycin (MMM) is a combination which has shown to be as active but less emetogenic than vincristine, adriamycin and cyclophosphamide regime (VAC) (Powles et al, 1991). When compared to CMF it was similarly well tolerated and with a comparable efficacy and toxicity spectrum (Jodrell et al, 1991). Both of these studies (Jodrell et al, 1991; Powles et al, 1991), however, showed significantly worse haematological toxicity occurring in patients on MMM regimes, with thrombocytopenia occurring in 34% of patients receiving MMM compared to 14% on CMF. In the MMM/VAC study, myelosuppression was greater in patients receiving MMM compared to those on VAC at day 21, i.e. the time for next treatment. There was also significantly greater grade 3 and 4 leucopenia and thrombocytopenia at day 21 following MMM than after the MM part of the regime, i.e. after only methotrexate and mitoxantrone had been administered. This finding led us to compare MMM with the MM regimen, since it was thought that mitomycin C may have been responsible for most of the haematological toxicity. No significant difference in objective response was found between these two regimes, which were well tolerated, but significantly less haematological toxicity and fewer dose delays and reductions were evident with MM (Stein et al, 1992).

Since the MM chemotherapy regime was relatively well tolerated, we decided to carry out a randomized trial to compare MM with CMF, incorporating comparisons of quality of life and side-effects on treatment.

*Received 2 November 1998*
*Revised 27 February 1999*
*Accepted 12 April 1999*

*Correspondence to:* RC Coombes

### PATIENTS AND METHODS

### Patient eligibility

All patients with cytologically or histologically proven locally advanced or metastatic breast cancer requiring treatment with cytotoxic chemotherapy were considered for entry into the trial. There were no age limits set for trial eligibility. Patients who had had prior treatment with any chemotherapeutic agents for locally advanced or metastatic breast cancer were excluded. Adjuvant chemotherapy had to have been completed more than 2 years prior to entry. Patients had to have measurable or evaluable lesions with documented progression within 2 months before entry into the study. Prior radiation to any of the present areas of measurable or evaluable disease also excluded the patient from the trial. Patients with psychiatric or addictive disorders, which would preclude obtaining informed consent or compliance with the quality of life studies, were considered ineligible. Further exclusion criteria were as follows: cardiac failure or significant dysrythmia, severe renal (blood urea nitrogen > 18 mmol $l^{-1}$) or hepatic impairment (bilirubin > four times normal), impaired bone marrow function (white cell count (WBC) $3.5 \times 10^9$ $l^{-1}$ or platelets < $150 \times 10^9$ $l^{-1}$), simultaneous endocrine therapy or endocrine therapy within 3 weeks of entry (when withdrawal response was considered to be possible) and evidence of an active second malignancy. The trial protocol was accepted by the ethical review board for this institution. All patients gave written consent to take part in the study.

### Treatment

The treatment schedules were as follows: the CMF regimen consisted of cyclophosphamide at a dose of 600 mg $m^{-2}$, methotrexate at 40 mg $m^{-2}$ and 5-fluorouracil at 600 mg $m^{-2}$, all administered intravenously (i.v.) on days 1 and 8. The cycle was repeated every 4 weeks for a total of six cycles. The MM regimen consisted of methotrexate at a dose of 30 mg $m^{-2}$ and mitoxantrone at 6.5 mg $m^{-2}$, both administered i.v. on day 1, repeating the cycle every 3 weeks for a total of eight cycles. Folinic acid was not used routinely. Dose modification was carried out for both CMF and MM regimens according to the levels of WBC and platelets. One hundred per cent of the dose was given if levels were WBC > $3.0 \times 10^9$ $l^{-1}$ and platelets > $100 \times 10^9$ $l^{-1}$. If the WBC level was 2.5–3.0 × $10^9$ $l^{-1}$ but platelets remained over $100 \times 10^9$ $l^{-1}$, the patient received 75% of the full dose. If the WBC level was < $2.5 \times 10^9$ $l^{-1}$ or platelets < $100 \times 10^9$ $l^{-1}$ a delay of 1 week was recommended. If blood urea was > 12 mmol $l^{-1}$, MM chemotherapy was not given. Methotrexate dose was reduced and folinic acid rescue given if the urea was between 9 and 12 mmol $l^{-1}$. MM was also withheld if serum bilirubin > 30 mmol $l^{-1}$ and AST were twice the upper limit of normal. Mitoxantrone dose was reduced if there was any evidence of impaired hepatocellular function. To prevent nausea and vomiting, dexamethasone (12 mg $day^{-1}$) and domperidone (60 mg $day^{-1}$) was suggested for all patients. If this failed, the addition of 5HT3 receptor antagonist to the dexamethasone was recommended.

### Study parameters and toxicity assessment

Patients were fully staged prior to randomization by clinical examination, chest X-ray, full blood count, serum urea, calcium, electrolytes and liver function tests. Liver ultrasound (optional if liver function tests were normal and liver was not palpable) and a limited skeletal survey consisting of lateral X-rays of the skull, cervical/lumbar spine and AP of the pelvis were also performed. Isotopic bone scans, computerized tomography scans and lung function tests were optional. An initial assessment was completed pre-treatment for each patient recording age, body surface area, menopausal status, performance status, disease-free interval and previous adjuvant chemotherapy/hormone therapy. Disease assessment was also carried out prior to initial chemotherapy recording haematology and sites and size of assessable disease. Patients encompassing the range of Karnofsky scores (EORTC, 1996) were included in the study, but this information was not recorded prospectively. However, only five patients were entered who had required hospitalization.

Response was defined using the UICC criteria (Hayward et al, 1977). Details of treatment, response, toxicity and clinical assessments of disease were made when chemotherapy was administered, i.e. 3-weekly for MM and 4-weekly for CMF. Radiological reassessment was carried out at week 12 (prior to course four CMF/course five MM) and week 28 (i.e. 4 weeks after completion of chemotherapy) or, if necessary, on early withdrawal. Toxicity was documented before giving each chemotherapy and was then recorded at weeks 12 and 24 as the worst since last assessment according to WHO criteria (WHO, 1979). Nausea/vomiting, diarrhoea, alopecia, skin rash, 'other toxicity' and haematology were all recorded specifically. After completion of treatment, patient follow-up continued at 6-monthly intervals, dates and causes of death being recorded where necessary.

### Quality of life

Quality of life (QOL) was measured according to the Hospital Anxiety and Depression Scale (HADS) (Zigmond and Snaith, 1983) and the Rotterdam Symptom CheckList (RSCL) (De-Haes et al, 1990), along with three additional 'patient satisfaction' questions asking, 'How satisfactory do you feel your hospital treatment has been?', 'What quality of life have you enjoyed in the past month?' and 'What quality of life did you enjoy before your illness?'. These last three questions were all measured on a four-point scale, possible responses to each being: extremely satisfactory, satisfactory, unsatisfactory or extremely unsatisfactory. Patients taking part in the QOL study were expected to complete a total of three assessments during their clinic visits pretreatment, at week 12 and week 24, or if appropriate on withdrawal from treatment if the patient consented. All available data were initially tabulated according to assessment visit but the main QOL analyses were based on patients having at least two assessments (i.e. an initial assessment pretreatment plus one other).

The most dissatisfaction noted during treatment for each of the three 'patient satisfaction' questions was compared with that noted at initial assessments. Data from the HADS and RSCL scales were analysed both as continuous scores and also categorized according to level of severity. HADS anxiety and depression were categorized as clinically abnormal state present (score 11+), borderline (8–10) or absent (0–7). The RSCL psychological scale was categorized as high (score 18+), borderline (14–17) or low (0–13) and the physical scale categorized similarly but with score groups 28+, 24–27, 0–23. Change in score or category from initial assessment to each treatment visit was calculated along with change to the maximum (i.e. most severe) score or category recorded during treatment. Combined analyses (not split by treatment group) were also used to

*© 1999 Cancer Research Campaign* 

**Table 1** Patient characteristics

| | CMF (*n* = 58) | MM (*n* = 58) | Total (*n* = 116) |
|---|---|---|---|
| Age | | | |
| <35 | 0 (–) | 3 (5%) | 3 (2%) |
| 35–<45 | 6 (10%) | 7 (12%) | 13 (11%) |
| 45–<55 | 14 (24%) | 8 (14%) | 22 (19%) |
| 55–<65 | 25 (43%) | 19 (33%) | 44 (38%) |
| 65–<75 | 9 (16%) | 11 (19%) | 20 (17%) |
| 75–<85 | 4 (7%) | 10 (17%) | 14 (12%) |
| *Median (range)* | *58 (37–80)* | *61 (28–84)* | *59 (28–84)* |
| Menopausal status | | | |
| Premenopausal | 6 (10%) | 10 (17%) | 16 (14%) |
| Post-menopausal | 52 (90%) | 48 (83%) | 100 (86%) |
| Histology – primary tumour type | | | |
| Infiltrating ductal | 49 (96%) | 48 (86%) | 97 (97%) |
| Infiltrating ductal & lobular | 2 (4%) | 1 (2%) | 3 (3%) |
| N/K | 7 | 9 | 16 |
| Sites of metastatic disease[b] | | | |
| Breast/chest wall | 24 (41%) | 23 (40%) | 47 (41%) |
| Lymph nodes | 10 (17%) | 15 (26%) | 25 (22%) |
| Liver | 19 (33%) | 22 (38%) | 41 (35%) |
| Bone | 34 (59%) | 31 (53%) | 65 (56%) |
| Previous adjuvant CT | 7 (12%) | 5 (7%) | 12 (10%) |
| Previous radiotherapy | 43 (74%) | 39 (67%) | 82 (71%) |
| Previous adjuvant tamoxifen or endocrine therapy[a] | 43 (74%) | 39 (68%) | 82 (71%) |
| Previous CT for advanced disease | 1 (2%) | 1 (2%) | 2 (2%) |

[a]One MM patient not known; [b]sites not mutually exclusive.

**Table 2** Best overall response

| | CMF (*n* = 58) | MM (*n* = 58) |
|---|---|---|
| CR | 2 (3%) | 3 (5%) |
| PR | 15 (26%) | 6 (10%) |
| NC | 15 (26%) | 15 (26%) |
| PD | 14 (24%) | 25 (43%) |
| 2+ courses of treatment given | 11 | 21 |
| <2 courses of treatment given | 3 | 4 |
| Not evaluable | 12 (21%) | 9 (16%) |

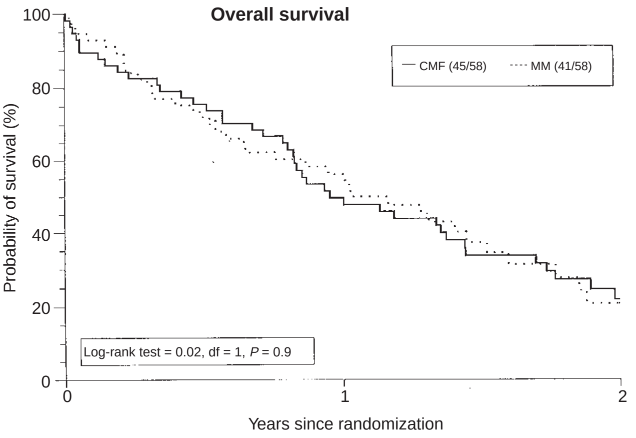

**Figure 1** CMF vs MM in Advanced Breast Cancer

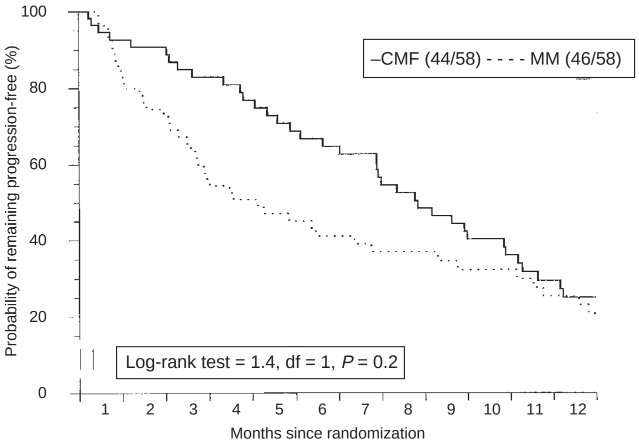

**Figure 2** CMF vs MM in Advanced Breast Cancer assess overall whether maximum toxicity during treatment was associated with HADS/RSCL quality of life score. Similar associations with the change in HADS/RSCL scores from initial assessment to that of maximum toxicity were also evaluated. For these analyses, if maximum toxicity was recorded at more than one visit, average QOL scores were calculated and, for change analyses, the difference between the average and initial assessment taken.

### Statistical methods

Data are presented as *n* (%) or median (range) as appropriate. Chi-squared tests were used to investigate associations between categorical variables. Linear trends in QOL scores across maximum toxicity levels were investigated using one-way analysis of variance. Log-rank tests (Peto et al, 1977) were used to compare the survival experience and time to progression, from randomization, between the two regimes. All patients, including those considered to be unevaluable for response, remained in the statistical analysis when comparing response rates between the two regimes and thus summary statements of response rates include all patients in the denominator unless otherwise indicated. Statistical analyses were conducted using SPSS version 4.1, apart from those assessing survival (for which in house, Fortran-based, software was used).

### RESULTS

One hundred and sixteen patients were randomized (58 to CMF and 58 to MM), between January 1992 and December 1996 all from Charing Cross Hospital, London. One patient was lost to follow-up after having received one course of treatment when she returned to Australia (see trial profile).

Initial treatment forms were received from all patients, and patient characteristics are summarized in Table 1. The median age of the patients randomized was 59 years (range 28–84). The majority (86%) were post-menopausal. Ninety seven per cent of primary

© 1999 Cancer Research Campaign

**Table 3** Experience of toxicity over treatment (patients with toxicity data available)

| Worst experience of: | WHO grading | CMF ($n$ = 48) | MM ($n$ = 47) | $P$-value for trend ($\chi^2_1$) |
|---|---|---|---|---|
| Nausea and vomiting | | | | |
| None | 0 | 19 (39%) | 26 (55%) | |
| Nausea | 1 | 12 (25%) | 9 (19%) | |
| Transient vomiting | 2 | 8 (17%) | 10 (21%) | |
| Vomiting – needed treatment | 3 | 8 (17%) | 1 (2%) | |
| Intractable vomiting | 4 | 1 (2%) | 1 (2%) | 0.08 |
| Diarrhoea | | | | |
| None | 0 | 39 (81%) | 44 (94%) | |
| Transient < 2 days | 1 | 5 (10%) | 2 (4%) | |
| Tolerable > 2 days | 2 | 4 (8%) | 1 (2%) | 0.07 |
| Alopecia | | | | |
| No change | 0 | 33 (69%) | 43 (91%) | |
| Minimal hair loss | 1 | 5 (10%) | 3 (6%) | |
| Moderate patchy loss | 2 | 6 (13%) | 1 (2%) | |
| Complete – reversible | 3 | 4 (8%) | 0 (–) | 0.002 |
| Anaemia (Hb) | | | | |
| 11 + g/dl | 0 | 24 (50%) | 32 (68%) | |
| 9.5–10.9 g/dl | 1 | 18 (38%) | 9 (19%) | |
| 8.0–9.4 g/dl | 2 | 4 (8%) | 5 (11%) | |
| 6.5–7.9 g/dl | 3 | 2 (4%) | 1 (2%) | 0.2 |
| Skin rash | | | | |
| No change | 0 | 47 (98%) | 46 (98%) | |
| Erythema | 1 | 0 (–) | 1 (2%) | |
| Dry desquamation | 2 | 1 (2%) | 0 (–) | 0.7 |
| Leucopenia (WBC) | | | | |
| $>4\times10^9$/l | 0 | 14 (29%) | 19 (41%) | |
| $3–3.9\times10^9$/l | 1 | 8 (17%) | 14 (30%) | |
| $2–2.9\times10^9$/l | 2 | 14 (29%) | 11 (24%) | |
| $1–1.9\times10^9$/l | 3 | 7 (14%) | 0 (–) | |
| $<1\times10^9$/l | 4 | 5 (10%) | 0 (–) | 0.001 |
| N/K | | 0 | 1 | |
| Platelets: | | | | |
| $>100\times10^9$/l | 0 | 42 (87%) | 41 (91%) | |
| $75–99\times10^9$/l | 1 | 3 (6%) | 3 (7%) | |
| $50–74\times10^9$/l | 2 | 1 (2%) | 1 (2%) | |
| $25–49\times10^9$/l | 3 | 0 (–) | 0 (–) | |
| $0–24\times10^9$/l | 4 | 2 (4%) | 0 (–) | 0.4 |
| N/K | | 0 | 2 | |

tumours were infiltrating ductal carcinoma. Most patients had multiple sites of metastatic disease. Over half had bone metastases, with other main sites comprising breast/chest wall, lymph nodes and liver (see Table 1). Ten per cent of patients had previous adjuvant chemotherapy, whereas 71% received previous radiotherapy and 71% previous adjuvant tamoxifen or endocrine therapy.

Twenty-one patients (18%) (12 CMF/9 MM) were deemed unevaluable for overall response assessment for the following reasons: two patients (CMF) were incorrectly randomized having previously received chemotherapy for advanced breast cancer and one patient (MM) had grossly abnormal liver function tests. Five patients died before treatment was given (all CMF). Seven patients died after one course of treatment prior to assessment of response (three CMF, four MM), and a further patient (CMF) stopped treatment due to toxicity after one course and changed treatment before assessment of response. Of the remaining five patients, one MM patient had a treatment deviation, one MM patient had a revised diagnosis of metastatic small bowel carcinoma, two MM patients had no assessable disease and one patient (CMF) stopped treatment with an intercurrent illness.

No statistically significant differences were seen between the two treatment groups with regards to survival (Figure 1), with 45/58 (78%) patients randomized to CMF and 41/58 (72%) of those randomized to MM being known to have died during the course of the study. Time to progressive disease (Figure 2) was also not found to be significantly different between the two treatment groups although there was a tendency towards a shorter time to progression on the MM regimen, with median time to progression in CMF patients was days compared to 109 days for patients randomized to MM, suggesting that MM was less effective than CMF (log-rank, $P$ = 0.2, see Discussion). Proportionality of hazards across time was investigated further given the shape, in particular, of the time to progression curves; the log-rank test being perhaps not the most powerful statistical test to use in situations of non-proportionality. However, when tested, formally, any deviation was not found to be severe enough to reach statistical significance.

On an intention to treat basis, lower percentages of patients receiving MM achieved a complete/partial response (15%, 9/58) compared to those on the CMF regimen (29%, 17/58) (95% confidence interval for difference in response rates –1%–29%, $P$ = 0.07, Table 2). Of the 95 patients evaluable for response, 37% of CMF patients achieved a complete/partial response compared to 18% of those receiving MM. Thus, there was a lower response rate for patients receiving MM chemotherapy.

Reasons for treatment being stopped were similar in the two treatment groups and were primarily due to progressive disease or

*© 1999 Cancer Research Campaign*

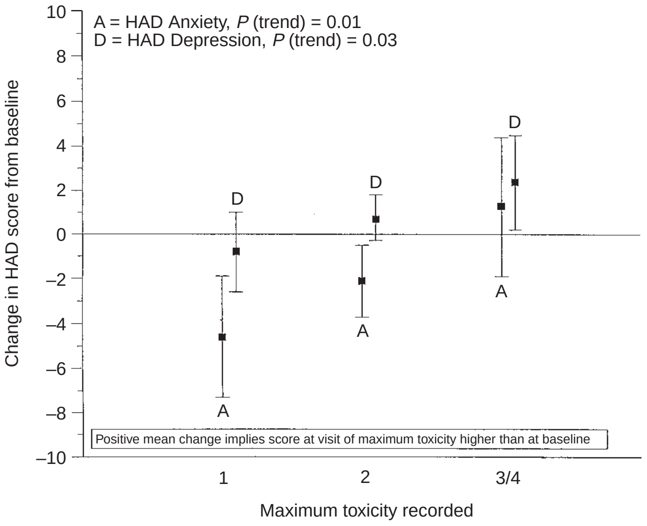

**Figure 3** Maximum toxicity over treatment and quality of life (HAD scores) Mean change (95% CI) in HAD score between baseline and the visit recording maximum toxicity, $n = 59$

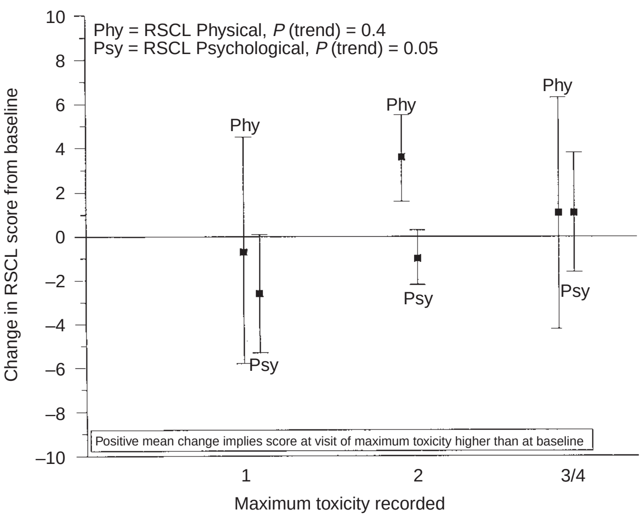

**Figure 4** Maximum toxicity over treatment and quality of life (HAD scores); Mean change (95% CI) in RSCL score between baseline and the visit recording maximum toxicity, $n = 59$

death. Twenty-nine patients receiving CMF (50%) and 18 MM (31%) completed treatment as per protocol ($P = 0.04$). Of the patients who completed treatment, 9/29 (31%) of those randomized to CMF completed without any deviations from treatment compared to 11/18 (61%) of those randomized to MM ($P = 0.04$). In the 20 CMF patients who had treatment deviations, 50% of deviations were as a result of haematological toxicity compared to none in the seven MM patients. Two toxic deaths were recorded, both patients receiving CMF, one being a result of haematological toxicity.

Toxicity data during treatment was available on 95 patients (48 CMF, 47 MM; Table 3). There was no evidence, in terms of statistical significance, of differences between the two regimes with regards to patient experience of nausea/vomiting, diarrhoea, anaemia, or skin rash. However, evidence of less severe leucopenia was apparent in MM patients ($\chi^2_1$ (trend), $P = 0.001$), with no patients recording WBC levels $< 2 \times 10^9$ $l^{-1}$ (grade 3 or above) compared to 12 (25%) patients receiving CMF. Five episodes of neutropenic sepsis were seen in patients receiving CMF compared with no patients in the MM arm of the study.

There was also evidence for less alopecia on the MM regimen ($P = 0.002$). Only three MM patients (6%) recorded minimal hair loss (grade 1) and 1 (2%) moderate patchy loss, with the remainder (91%) having no change. In comparison, 15 (31%) patients receiving CMF recorded some level of alopecia. Five of these had minimal hair loss (grade 1), six moderate patchy loss (grade 2) and four complete (reversible – grade 3). Severity of 'other toxicity' reported was very similar between the two treatment groups, the more commonly reported forms being mucositis/oral discomfort (most often at grade 1 or 2 severity), fatigue/malaise/headaches and constipation, the latter two usually reported at grade 1 severity.

Due to reduced patient numbers following early study termination (see further comment in Discussion), comprehensive evaluation of QOL data was not possible with any satisfactory level of power. However, data available were investigated in an attempt to establish whether any general trends were apparent. Eighty-eight patients were well enough to complete the first QOL assessment. However, overall 28 patients completed only one assessment, 25 completed two and only 35 (26 CMF and nine MM) had the desired complete set at initial assessment, 12 weeks and 24 weeks. One patient completed two QOL assessments at 12 weeks and 24 weeks, but was thereafter unevaluable in terms of change from initial visit, leaving 59 patients evaluable with regards to change scores.

Of the 28 patients who only completed one QOL assessment, 19 did not complete the 12-week assessment as they had come off treatment because of toxicity, progressive disease or death. The remaining nine patients failed to complete the questionnaire, either because they refused (six) or did not receive the form (three).

Analysis of QOL data by assessment visit showed neither evidence of differences between treatment groups, nor of differences between weeks 12 and 24 or from patients withdrawing early. The 'on treatment data' responses obtained were therefore combined across visits into summary measures of 'most dissatisfaction' and maximum score and evaluated in terms of change from initial assessment.

HADS and RSCL scales did not reveal evidence of differences between the two groups in terms of maximum change from initial assessment, whether analysed by categorical change in state or using the data in continuous form. Only five patients (three CMF, two MM) showed any sign of being dissatisfied with the hospital treatment they had received, and no statistically significant differences were seen between the two regimes in terms of maximum change in satisfaction or QOL from initial assessment, results thus reflecting results from HADS and RSCL scales. However, some association between QOL and maximum toxicity experience was apparent overall, in the 59 patients with two or more QOL assessments, with mean changes in HADS anxiety/depression and RSCL psychological scores from initial assessment to the visit at which maximum toxicity was noted all showing some tendency to increase with rising maximum toxicity ($P = 0.01$, $P = 0.03$, $P = 0.05$ respectively; Figures 3 and 4).

We carried out an analysis of the treatment patients received after CMF or MM chemotherapy. In the CMF group, 21 patients had one further course and five had two further forms of

 © 1999 Cancer Research Campaign chemotherapy: 12 received epirubicin and five Taxotere; nine patients had MM. In the MM group, 21 patients had further treatment with chemotherapy: five received epirubicin and five Taxotere; five patients had CMF. Six received 5-fluorouracil, epirubicin and cyclophosphamide chemotherapy.

### DISCUSSION

Our study shows that the methotrexate/mitoxantrone (MM) regimen is likely to be less active than CMF, but MM may be a useful first-line chemotherapy schedule for the palliation of patients with advanced or metastatic breast cancer in whom side-effects should be avoided. First, no patients receiving MM suffered from severe leucopenia (WBC $< 2 \times 10^9\ l^{-1}$), compared to 12 on CMF. Secondly, only 8% of MM patients developed alopecia, with only a single MM patient complaining of moderate hair loss compared to 31% of CMF patients. Potential drawbacks of the MM regimen include (a) a reduced response rate, and (b) a shorter time to progression overall, although in this randomized study no statistically significant differences were found in response rate, survival or progression free survival between MM and CMF (Table 2, Figures 1 and 2). We also observed that 55% of CMF and 41% of MM patients achieved either CR/PR/NC and the number of patients who died shortly after chemotherapy because of progressive disease was similar in both treatment groups. These features suggest that, provided the chemotherapy is substituted for a more aggressive regimen on clinical evidence of disease progression, the MM regimen does not jeopardize survival.

Although no significant differences in response rate, survival or times to progression were found in this study, it must be noted that statistical power was low. The trial was originally planned to recruit 326 patients to give an 80% power (two-sided $\alpha = 0.05$) to detect a 15% absolute difference in response rates assuming a 30% response in patients treated with MM. However, due to poor accrual, the study was prematurely terminated on the advice of an Independent Data Monitoring Committee.

Our overall response rate with CMF compares well with the protocol used by Tannock et al (1998). However, those authors highlight the variations that exist in response rates between institutions using similar regimes and comment that they are often not meaningful as patient selection and treatment policy differ, such as in the use of endocrine therapy as first-line in metastatic disease. This is exemplified by comparing this study, in which the patients were heavily pretreated with hormonal agents (74% and 64% with CMF, and 68% and 60% with MM in the adjuvant and advanced treatment settings respectively), with other CMF trials which have either not recorded previous hormone treatment, not specified in which setting it was previously given, or had lower numbers of previously treated patients (Bull et al, 1978; Tormey et al, 1982; Aisner et al, 1987; Cummings et al, 1995). Another trial comparing CMF with MMM did have hormone pretreatment levels of 70%, i.e. approaching our own, and had a CMF response rate of 60%, but adjuvant chemotherapy was an exclusion (Jodrell et al, 1991).

We obtained a comparable median survival with both regimes in our group of patients and the survival observed is similar to other recent studies (Clavel and Catimel, 1993, Stewart et al, 1997). It was our practice, during the course of the study, to treat patients with a sequence of an anthracycline, and subsequently a taxane after relapse or failure to respond to CMF or MM. This is a practice that is followed in many cancer centres. At present the aim of most trials in advanced breast cancer involving the use of newer agents, e.g. the taxanes and high-dose trials is to improve response and, hopefully, also survival. In the case of advanced breast cancer there is little evidence that improvement in CR rate will manifest in significantly increased long-term survival and metastatic breast cancer remains essentially incurable. More recent trials, however, have shown some statistical response advantage with anthracycline containing regimes, but not all have shown a statistical survival or response duration advantage (Muss et al, 1978; Brickner et al, 1984; Tormey et al, 1984; Smalley et al, 1983). Recently A'Hern et al (1993) used summary statistics on the trials that included doxorubicin in the Cooper-type regimes (Cooper et al, 1969) to arrive at a median survival improvement of a fifth. Such small improvements in survival and responses do not necessarily translate into enhanced QOL and other studies have demonstrated that there is no conclusive evidence of survival advantage although response rates were improved (Gradishar et al, 1996).

Our study is also one of a small number of breast cancer trials to examine QOL using standardized scales, although our experience underlines the difficulties in successfully obtaining serial QOL estimates in this patient population. A recent study compared QOL in intermittent and continuous administered chemotherapy schedules. Although survival was similar in both arms of the study, QOL was better in patients receiving treatment continuously compared to those receiving it in 3-monthly blocks of treatment (Coates et al, 1987). Indeed, we could find no recent breast cancer trial that has used the HADS and RSCL scales which have been recommended by Maguire and Selby, 1989). Although we were not in a strong position to evaluate differences between the two groups because of low power due to premature study termination and the small number of patients who were able to complete the QOL study, we were able to show trends towards deteriorating QOL with rising maximum toxicity overall. Other authors have discussed the relationship between toxicity and QOL, reporting that the latter is usually although not always related to the former (Payne, 1992).

In conclusion, the MM regime is a regime which could be considered to be better tolerated than the standard CMF regimen for metastatic breast cancer. The limitations of this trial must be acknowledged, but with careful patient management there may be potential for MM to be considered as a first-line treatment, particularly in those who are frail or for those in whom leucopenia is undesirable.

### ACKNOWLEDGEMENTS

We thank the CRC for programme grant support. Our thanks also to referring clinicians, especially Dr R Phillips and also Mrs E Woods and Lilly Oncology.

### REFERENCES

Aisner J, Weinberg V, Perloff M, et al (1987) Chemotherapy versus chemoimmunotherapy (CAFV CAFVP v CMF IMER) for metastatic carcinoma of the breast. A CALGB study. *J Clin Oncol* **5**: 1523–1533

Bull JM, Tormey DC, Shou-Hua L, et al (1978) A randomized comparative trial of adriamycin versus methotrexate in combination drug therapy. *Cancer* **41**: 1649–1657

Canellos GP, De Vita VT, Gold GL, et al (1976) Combination chemotherapy for advanced breast cancer: response and effect on survival. *Ann Int Med* **84**: 389–392

Clavel M and Catimel G (1993) Breast cancer: chemotherapy in the treatment of advanced disease. *Eur J Cancer* 29A **4**: 598–604

Cummings FT, Gelman R and Horton J (1995) Comparison of CAF versus CMFP in metastatic breast cancer: Analysis of prognostic factors. *J Clin Oncol* **3**: 932–940
De-Haes JC, van-Knippenberg FC, Neijt JP (1990) Measuring psychological and physical distress in cancer patients: structure and application of the Rotterdam Symptom Checklist. *Br J Cancer* **62**: 1034–1038
EORTC: A practical guide to EORTC Studies. 1996 February
Greenspan EM (1966) Combination cytotoxic chemotherapy in advanced disseminated breast carcinoma. *J Mt Sinai Hosp* **33**: 1–27
Hayward JL, Rubens RD, Carbone PP et al (1977) Assessment of response to therapy in advanced breast cancer. *Br J Cancer* **35**: 292–298
Honig SF (1996) Treatment of metastatic disease: hormonal therapy and chemotherapy. In: *Diseases of the Breast*, Harris JR, Lippman MC, Morrow M, et al (eds), pp. 669–734. Lippincott: Philadelphia
Jodrell DI, Smith IE, Mansi JL et al (1991) A randomised comparative trial of mitoxantrone/methotrexate/mitomycin C (MMM) and cyclophosphamide/methotrexate/5 FU (CMF) in the treatment of advanced breast cancer. *Br J Cancer* **63**: 794–798
Maguire P and Selby P (1989) Assessing quality of life in cancer patients. *Br J Cancer* **60**: 437–440
Mouridsen H (1992) Systemic therapy of advanced disease. *Drugs* **44**: 17–28
Payne SA (1992) A study of quality of life in cancer patients receiving palliative chemotherapy. *Soc Sci Med* **35**: 1505–1509
Peto R, Pike MC, Armitage P et al (1977) Design and analysis of randomised clinical trials requiring prolonged observation of each patient. Part 2 Analysis and examples. *Br J Cancer* **35**: 1–39
Powles TJ, Jones AL, Judson IR et al (1991) A randomised trial comparing combination chemotherapy using mitomycin C, mitoxantrone and methotrexate (3M) with vincristine, anthracycline and cyclophosphamide (VAC) in advanced breast cancer. *Br J Cancer* **64**: 406–410
Smalley RV, Lefante J, Bartolucci A et al (1983) CAF v CMFVP in patients with advanced breast cancer. *Breast Cancer Teat Res* **3**: 209–210
Stein RC, Bower M, Law M et al (1992) Mitoxantrone and methotrexate chemotherapy with and without mitomycin C in the treatment of advanced breast cancer: A randomised clinical trial. *Eur J Cancer* **28A**: 1963–1965
Stewart DJ, Evans WK, Shepherd FA et al (1997) Cyclophosphamide and fluorouracil combined with mitoxantrone versus doxorubicin for breast cancer: superiority of doxorubicin. *J Clin Oncol* **15**: 1897–1905
Tannock IF, Boyd NF, DeBoer G et al (1988) A randomised trial of two dose levels of cyclophosphamide, methotrexate and fluorouracil chemotherapy for patients with metastatic breast cancer. *J Clin Oncol* **6**: 1377–1387
Tormey DC, Gelman R, Band PR et al (1982) Comparison of induction chemotherapies for metastatic breast cancer. An Eastern Cooperative Oncology Group Trial. *Cancer* **50**: 1235–1244
World Health Organisation (1979) *WHO Handbook for Reporting of Cancer Treatment* .WHO: Geneva
Zigmond AS and Snaith RP (1983) The hospital anxiety and depression scale. *Acta Psychiatr Scand* **67**: 361–363

 *© 1999 Cancer Research Campaign*
